# Gastroparesis: The Complex Interplay with Microbiota and the Role of Exogenous Infections in the Pathogenesis of the Disease

**DOI:** 10.3390/microorganisms11051122

**Published:** 2023-04-25

**Authors:** Francesco Vito Mandarino, Emanuele Sinagra, Alberto Barchi, Maria Chiara Verga, Daniele Brinch, Dario Raimondo, Silvio Danese

**Affiliations:** 1 Division of Gastroenterology and Gastrointestinal Endoscopy, San Raffaele Hospital, Vita-Salute San Raffaele University, 20132 Milan, Italy; 2 Gastroenterology & Endoscopy Unit, Fondazione Istituto G. Giglio, Contrada Pietra Pollastra Pisciotto, 90015 Cefalù, Italy; 3 Gastroenterology and Digestive Endoscopy Unit, ASST Cremona, Viale Concordia 1, 26100 Cremona, Italy; 4 Gastroenterology & Hepatology Section, PROMISE, University of Palermo, 90127 Palermo, Italy

**Keywords:** functional gastrointestinal disorders, gut–brain axis disorders, gastroparesis, microbiota, infections

## Abstract

Gastroparesis (GP) is a disorder of gastric functions that is defined by objective delayed gastric emptying in the absence of mechanical obstruction. This disease is characterized by symptoms such as nausea, post-prandial fullness, and early satiety. GP significantly impacts patients’ quality of life and contributes to substantial healthcare expenses for families and society. However, the epidemiological burden of GP is difficult to evaluate, mainly due its significant overlap with functional dyspepsia (FD). GP and FD represent two similar diseases. The pathophysiology of both disorders involves abnormal gastric motility, visceral hypersensitivity, and mucosal inflammation. Moreover, both conditions share similar symptoms, such as epigastric pain, bloating, and early satiety. The latest evidence reveals that dysbiosis is directly or indirectly connected to gut–brain axis alterations, which are the basis of pathogenesis in both FD and GP. Furthermore, the role of microbiota in the development of gastroparesis was demonstrated by some clinical studies, which found that the use of probiotics is correlated with improvements in the gastric emptying time (GET). Infections (with viruses, bacteria, and protozoa) represent a proven etiology for GP but have not been sufficiently considered in current clinical practice. Previous viral infections can be found in about 20% of idiopathic GP cases. Moreover, delayed gastric emptying during systemic protozoal infections represents a huge concern for compromised patients, and few data exist on the topic. This comprehensive narrative review analyzes the relationship between microorganisms and GP. We explore, on the one hand, the correlation between gut microbiota dysbiosis and GP pathogenesis, including treatment implications, and, on the other hand, the association between exogenous infections and the etiology of the disease.

## 1. Introduction

### 1.1. Gut Microbiota Principles

The human microbiota is one of the densest and most quickly developing ecosystems [1]. The term “microbiota” usually defines the assemblage of living microorganisms present in a defined environment [2]. However, as phages, viruses, plasmids, prions, viroids, and free DNA are usually not considered living microorganisms [3], they do not belong to the microbiota. The term microbiome, as originally postulated by Whipps et al. [4], includes not only the community of microorganisms, but also their “theatre of activity” [5].

In healthy conditions, microbiome bacteria interact with the epithelial barrier, with immune cells modulating their responses in addition to influencing local metabolism through their own metabolites, thereby maintaining homeostasis [6,7]. For example, the normal gut microbiome imparts specific functions in host nutrient metabolism, xenobiotic and drug metabolism, the maintenance of the structural integrity of the gut mucosal barrier, immunomodulation, and protection against pathogens [8]. Therefore, an imbalance of the gut microbiome due to the use of antibiotics or bacterial translocation can lead to the development of diseases [7,9].

The human gastrointestinal tract is one of the largest storage spaces for microbes in the body and contains both commensal and pathogenic microbial species [5,10].

Gut microbiota alterations in the small bowel and colon have been observed extensively in patients with Functional Gastrointestinal Disease (FGID) [11,12]. Certain studies showed that the gut microbiota profile of Irritable Bowel Syndrome (IBS) patients significantly differs from that of healthy subjects. A recent meta-analysis showed that IBS patients presented higher levels of bacteria belonging to the family Enterobacteriaceae, namely, the species *Escherichia coli* and genus *Enterobacter*, which contain opportunistic and virulent strains, as well as lower symbiont bacteria of the genera *Bifidobacterium* and *Lactobacillus* compared to healthy controls [13].

Indeed, gut microbiota alterations influence gut motility, the intestinal gas profile, gut immune and intestinal barrier functions, visceral sensation, the neuroimmunoendocrine interface, and the gut–brain axis [14].

Taken together, this breakthrough evidence indicates that gut microbiota alteration is a common finding in FGIDs and suggests that reforming the gut microbiota composition might be a potential treatment for FGIDs [11].

### 1.2. Gastroparesis: Definition, Pathology, and Clinical Aspects

Gastroparesis (GP) is a disorder of gastric motility, characterized by delayed gastric emptying in the absence of mechanical obstruction [15]. The symptoms of this disorder are non-specific and include nausea, vomiting, bloating, early satiety, and abdominal pain [16].

GP has a significant impact on patients’ quality of life and contributes significantly to healthcare expenses for families and society [17,18]. However, data on the epidemiology of GP are still unknown due to the overlap between GP symptoms and those of other functional diseases, especially functional dyspepsia (FD) [19]. In general practice, GP has an estimated prevalence of 24.2 per 100,000 people in the United States of America (USA) and 13.8 per 100,000 people in the United Kingdom (UK) [20,21].

One of the largest population-based studies in Minnesota in the United States found that patients with gastroparesis had a mean age of onset 44 years, and the incidence of definite gastroparesis increased significantly with advancing age, with a peak incidence of 10.5 per 100,000 in patients ≥ 60 years of age. The incidence and prevalence of gastroparesis in women were four times higher than those in men [20].

Since FGID and gastroparesis (GP) overlap, there is a subgroup of FGID that features dysmotility-like symptoms and mildly delayed gastric processes. On the other hand, true gastroparesis with invalidating upper GI symptoms and moderately to severely delayed emptying is much more pronounced than FD and is, therefore, a different entity.

The etiology of GP is varied. This disorder is associated with diabetes (in about one-third of patients), post-surgical conditions (surgery involving damage of the vagus nerve, including esophagectomy), autoimmune diseases (Systemic Lupus Erythematosus and sclerodermia) and neurological conditions (Parkinson’s disease and multiple sclerosis). Moreover, certain bacterial, viral, and protozoal infections are associated with the development of GP. However, in more than 50% of cases, a single cause is not identified (idiopathic GP) [22].

Previous studies, mainly based on cutaneous electrogastrography (EGG), have revealed that a high proportion of patients with gastroparesis had abnormalities in gastric myoelectrical activity [23,24]. However, EGG is critically limited by its summative nature and incomplete sensitivity. Recent discoveries related to histopathological alterations related to gastroparesis have reinvigorated interest in the electrical diagnostic of the disease. In this regard, the advent of high-resolution (HR; multi-electrode) mapping has been a key advance. Evidence suggests that gastric arrhythmias, defined as “disorders of initiation” or “disorders of gastric conduction”, probably linked to the depletion of interstitial Cajal cells, may play a role in symptom generation in gastroparesis [25], particularly in contributing to chronic nausea [26,27]. New data are needed to determine whether arrhythmias can be meaningfully treated in a way that improves organ function and quality of life in patients [27].

The gold standard examination for diagnosing GP is Gastric Emptying Study (GES) with a solid radiolabeled meal. A consensus statement established that the objective discriminating feature of GP is documentation of gastric retention >60% and >10% at 2 and 4 h, respectively [28]. It is essential that emptying be continued for at least 4 h after meal ingestion. Several methods exist to measure gastric emptying, including wireless motility capsules and stable isotope (13C spirulina) breath tests. However, the reproducibility is poor to moderate, and the tests are time-consuming and costly.

The first line of treatment includes dietary advice and pharmacological therapy. Dietary guidance is a cornerstone in support for GP patients. Patients with severe GP become malnourished and lose weight such that additional oral feeding or enteral tube feeding is indicated. First-line pharmacotherapy includes domperidone, metoclopramide, and erythromycin, while second-line pharmacotherapy includes prucalopride, acotiamide, motilin agonists, and neuromodulators. Cases not responding to pharmacotherapy should be considered for enteral tube feeding, PEG-J placement with enteral feeding, or endoscopic therapy [29].

G-POEM (Gastric Peroral Endoscopic Myotomy) is the preferred endoscopic treatment for refractory GP [30]. Since the clinical success of G-POEM is approximately 61% at one-year post-treatment [31], pre-operative GES parameters such as Intragastric Meal Distribution (IMD) are correlated with the outcome and can guide clinicians in the accurate selection of patients [32].

In recent decades, advances have been made in understanding the physiological and pathological mechanisms associated with GP, although many aspects remain unclear.

Histological alterations in the gastric muscle and myenteric plexus associated with the disease include the reduction and morphological alteration of Cajal cells, alterations of inhibitory (neuronal nitric oxide synthase, VIP, neuropeptide Y) and excitatory (acetylcholine and substance P) nerve fibers of the enteric nervous system (ENS), and inflammatory infiltrate [33].

Substance P deficiency has been shown to result in the loss of interstitial of Cajal cells and decreased neuronal nitric oxide synthase in patients with type II diabetes [34].

In an experimental setting, it was found that diabetic rats and rats with diabetic gastroparesis decreased levels of substance P compared to the control group. Additionally, rats with diabetic gastroparesis had significantly lower levels of neuropeptide Y than both diabetic rats and the control group. Interestingly, unlike diabetic rats, they did not show an increase in protein levels of the neuropeptide receptor Y1 (NPY-Y1) in the smooth muscle of pylorus. Based on these findings, depleted neuropeptide Y and the absence of NPY-Y1 upregulation may be involved in gastroparesis development [35].

Recent research suggested that the microbiota may play a role in the development and progression of GP. In this review, we examine the current state of knowledge on the relationship between GP and gut microbiota, including treatment implications. In addition, we analyze the infections that are associated with disease onset.

## 2. Microbiota and Gastroparesis

### 2.1. Bacterial “Milieu” in Functional Dyspepsia–Gastroparesis: Experimental Evidence and the Role of Dysbiosis

FD and GP are two gastric neuromuscular disorders that share similar features [36]. Pathophysiologically, both are characterized by abnormal gastric motility, mainly impaired gastric accommodation, and visceral hypersensitivity [37]. It was demonstrated that gastric alterations in these two conditions are triggered by central brain processing and signaling distortions, resulting in gut–brain axis alteration [38]. Thus, psychosocial issues such as stress represent some of the most influential factors that facilitate the onset of these symptoms [39].

The role of the microbiota in the pathogenesis of FGID was also hypothesized. Gastric bacterial overgrowth seems to be a rare situation and may be associated with short- and long-term complications; however, the link with gastroparesis has never been elucidated.

The duodenum has emerged as a key player in the development of upper gastrointestinal disorders such as FD and GP. Duodenal micro-inflammation is characterized by increased mucosal T-cell homing and higher mucosal permeability with the recruitment of eosinophils and mast cells via Th2 immune responses due to higher mucosal antigen presentation. This phenomenon is one of the pathological signatures of gut–brain axis alterations [40].

Our understanding of the extent to which duodenal dysbiosis contributes to the development of duodenal micro-inflammation and the distortion of gastric feedback, ultimately leading to the development of FD/GP, remains incomplete.

The duodenal microbiota is characterized by a relatively small abundance of bacteria (ranging from 10^1^ to 10^3^ bacteria/ml) and has a different taxonomic composition compared to other sites in the gut, with a greater prevalence of aerobic Gram-positive bacteria as opposed to obligate anaerobes or Gram-negative bacteria found in the remnant small intestine and colon [41].

Studies investigating modifications of the gut microbiota following exogenous infections have been conducted to shed light on the effects of post-infectious dysbiosis on the onset of functional gastrointestinal disorders [42,43]. Enteric infections, particularly those caused by Gram-negative bacteria, produce toxins that activate the immune system both locally and systemically, thereby altering the eubiotic gastro-duodenal “milieu” and provoking symptoms of post-prandial distress and nausea, which are characteristic of FD and GP, as shown in a recent study by Talley et al. [44].

Small intestinal bacteria overgrowth (SIBO) is defined as an increased number of bacteria in the small intestine. However, its association with GP/FD remains unclear.

Gurusamy et al. investigated the association between SIBO and FD in a recent meta-analysis and found that patients suffering from FD were 4.3 times more likely to have SIBO compared to the controls [45].

The correlation between GP and SIBO could be explained by the hypothesis that delayed gastric emptying can provoke bacterial overgrowth in the small intestine.

Reddymasu et al. performed a cohort study to measure the prevalence of SIBO in a cohort of gastroparetic patients and identify GP predictors of SIBO development. The authors enrolled 50 patients affected by GP with a predominance of abdominal pain and bloating and showed that 30/50 (60%) subjects had a positive glucose breath test for SIBO. Particularly, SIBO was more likely (p < 0.001) under a greater duration of gastroparetic symptoms (mean: 5 years). However, gastric emptying was not different between the SIBO and non-SIBO groups (p > 0.05) [46].

George et al. aimed to determine symptoms of SIBO in gastroparetic patients. The authors enrolled 201 gastroparetic patients, among whom 79/201 (39%) had a positive lactulose breath test for SIBO, and, interestingly, found that the hydrogen level increased to only >20 ppm above baseline by 90 min, which represented one of the three criteria for diagnosis and was correlated with typical gastroparetic symptoms (bloating and excessive fullness during and after meals) [47].

The breath test is a non-invasive and simple option for diagnosing SIBO. However, this test can suffer from false positives and negatives (with specificity of 44–100% and sensitivity of 17–89%) [48,49]. Moreover, as postulated by Clarke [50], this test is an unsuitable method for diagnosing SIBO in patients with GP since the results can be influenced by gut motility. Given the limitations of the breath test for the diagnosis of SIBO, published studies should be interpreted with caution.

Recently, Calderon et al. examined the association between GP and SIBO diagnosed through a culture of small bowel aspirates, thereby overcoming the limitations of a breath test. Among research participants who underwent enteroscopy for the evaluation of SIBO, the authors identified 73 subjects who underwent GES via scintigraphy. Twenty-nine participants were diagnosed with SIBO and 44 without SIBO, while 33 (45%) had evidence of GP. Remarkably, no significant association was found between SIBO diagnoses and delayed gastric emptying when using scintigraphy. The authors concluded that the microbiological diagnosis of SIBO is not associated with gastric emptying [51].

Further studies are needed to clarify the correlation between delayed gastric emptying and duodenal bacterial overgrowth and dysbiosis.

The development of symptoms of GP and FD is associated not only with an increase in bacterial overgrowth in the duodenum (as mentioned earlier for SIBO) but also with alterations of microbial composition [52].

The composition of duodenal Mucosal associated Microbiota (MAM) has been intensively studied using advanced DNA sequencing techniques [53]. MAM seems to have little correlation with luminal or fecal microbiota, remains relatively protected from contamination, and reflects its own features.

Zhong et al. analyzed the MAM of nine patients affected by FD to assess differences in MAM composition between FD patients and controls. The authors found that the *Streptococcus* genus predominated in both FD patients and the controls but with an inverse relationship between the relatively higher abundance of *Streptococcus* and the anaerobic genera *Prevotella*, *Veillonella*, and *Actinomyces*, which were more decreased in FD than in the controls (p < 0.05). The authors highlighted an inverse correlation between the total bacterial load (expressed in bacteria/mL) and disease severity, finding that as the total bacterial load increased, bacterial diversity decreased [54].

The gastroduodenal impairment pivotal in the development of GP and FD appears to be linked to different pathophysiological alterations in gastric and duodenal functions, primarily characterized by duodenal acid exposure and bile acid output. Presumably, alterations in bile and acid cycles lead to the development of FD/GP through changes in the microbiota [55].

Simren et al. explored the effects of gastroduodenal acid exposure on symptom onset and found that after external irrigation of the distal stomach with an acid liquid compound versus water, antral motility decreased, while duodenal contractions increased. These alterations were reflected in the aggravation of dyspepsia [56].

The use of proton pump inhibitors (PPIs) was shown to be useful in evaluating how alterations in duodenal acid exposure are associated with changes in the duodenal microbiota.

Two different research studies, one conducted by Jackson et al. and another conducted by Imhann et al., reported the presence of MAM distortion in individuals who had been using PPI therapy for extended periods of time [57,58]. The authors reported a noticeable increase in the prevalence of oral-like microbiota, with genera such as *Enterococcus*, *Streptococcus*, and *Staphylococcus* being more prominent in the gut microbiota of long-term PPI users compared to non-PPI users. The studies also suggested the presence of potentially pathogenic species such as Escherichia coli in the gut microbiota of long-term PPI users.

Current data related to the impact of PPIs on the duodenal microbiota composition [59,60] have produced speculation on their actual usefulness in the treatment protocols for FD and even GP [29].

The shift towards an oral-like or fecal-like microbiota composition was addressed as the potential mechanism of persistence of gastroparetic/dyspeptic symptoms in a sub-group of patients [61].

Wauters et al. recently conducted a prospective study on dyspeptic FD patients undergoing PPI therapy and found a significant change in the MAM during long-term PPI therapy, specifically in the abundance of *Porphyromonas* and *Streptococcus*. However, a subgroup analysis of symptom–responder patients revealed relative stability in the MAM during short-term treatment [62]. This finding suggests that short-term PPI therapy may be a viable option for managing immediate symptoms, with subsequent discontinuation of treatment to avoid a dysbiotic shift in the duodenal microbiota profile.

Given the existence of dysbiosis induced by PPIs and the aforementioned probable overlap with SIBO, it was hypothesized that a course of rifaximin, an intestinal-selective antibiotic with eubiotic effects, may be empirically useful for relieving dyspeptic symptoms [63]. A recent randomized controlled trial (RCT) demonstrated the moderate superiority of rifaximin over the placebo in terms of the global symptom response (78% versus 52%) at 8 weeks, as well as an improvement in post-prandial fullness at 4 weeks [64]. These findings support the hypothesis that the duodenal microbiota may be closely correlated with the onset of dyspeptic symptoms and the persistence of dyspeptic symptoms and GP.

Concerning bile acid output, it is widely accepted that bile acids may have an anti-microbial effect in the gut, reducing the total bacterial load and microbiota diversity [65]. A recent study by Beeckmans et al. confirmed a decreased concentration of bile salts in the duodenum of FD patients during the fasting state and suggested that a higher abundance of primary versus secondary bile acids could be indirectly correlated with increased duodenal permeability and altered intestinal barrier function [66].

Currently, few data are available on the key role of the duodenal microbiota in the pathogenesis of GP and its link to duodenal microinflammation [67,68]. However, it seems highly probable that the complex duodenal bacterial environment may hold several not-yet-elucidated secrets regarding duodenogastric motility [69].

### 2.2. Novel Insights in Treatment

Probiotics are live microorganisms that provide health benefits when consumed in adequate amounts [70]. Probiotics can be found in fortified foods, such as yogurt and smoothie drinks, and in supplements. Research has confirmed the benefits of probiotic use in alleviating a host of GI ailments, such as chronic intestinal inflammation, irritable bowel syndrome, diarrhea, and constipation [71]. Some beneficial effects of probiotics have been known for many years, but data on the effects of probiotics in functional GI disorders remain limited [72].

Some specific bacterial probiotic strains have been shown to improve symptom severity and abdominal pain in IBS patients [73,74,75,76,77,78,79,80,81,82,83,84,85,86,87,88,89,90,91,92], although their mechanisms of action remain unclear. There are some species that appeared to be more effective than others in clinical trials, such as *Bifidobacterium* species (*B. infantis* 3564 and *B. bifidum* MIMBb75) 172, 176, and 178 and Lactobacillus species (*Lactobacillus acidophilus*-SDC 2012, 2013, *L. paracasei* B2106, *L. plantarum* 299 V, and *L. rhamnosus* GG) [93]. These probiotics appear to be effective in reducing abdominal pain and discomfort in adults and children (*L. rhamnosus* GG) [93]. Probiotics act by improving rhythmic colonic contractions. They also colonize the bowels and modify the balance of the existing intestinal environment and its metabolic activity, thus offering benefits to the host [93]. Finally, the products of lactic acid bacteria fermentation influence local and distal motor events [94]. Based on the aforementioned evidence, it was hypothesized that probiotics are effective in treating GP. Some clinical studies have focused on this specific effect, as outlined in Table 1. Wang et al. investigated the effects of capsules containing three species of Lactobacillus on gastric emptying functions by using a crossover placebo-controlled clinical trial. The 15 healthy participants recruited were divided into two groups, with one group of seven people aged between 20 and 40 years and another group of eight people aged between 41 and 60 years. In this study, c-99m scintigraphy was performed at the beginning of weeks 0, 3, and 6 to determine the gastric emptying rate. All time–activity curves were constructed, and the half-time of gastric emptying (GEt1/2) was calculated for the same subject as a point of comparison. A comparison of the two groups indicated the positive effect of probiotic capsules on the 41- to 60-year-old participants (p = 0.013) but not on the 20- to 40-year-old participants. There was no significant statistical difference between the two groups in the period of treatment with placebo. This early-stage trial indicated that the multi-strain Lactobacillus capsule is safe, and the results provided some evidence that it accelerated gastric emptying in healthy adults older than 40 years of age and may offer, by implication, a therapeutic approach in future trials for pathological gastric emptying delay, especially in diabetic GP [94]. Ringel-Kulka et al. studied the clinical efficacy of *Lactobacillus acidophilus* and *Bifidobacterium lactis* in non-constipation FGIDs through a double-blind, placebo-control clinical trial performed on 60 patients. The authors concluded that administration of these probiotics twice a day improved symptoms of bloating in patients with FGIDs [95]. Indrio et al. investigated the putative effects of probiotics on the frequency of regurgitation and GET (gastroesophageal transit) in infants with gastroesophageal reflux. The authors enrolled 42 symptomatic children who were randomly selected to consume Lactobacillus reuteri or placebo every day for 30 days. The authors concluded that in infants with gastroesophageal reflux disease, L. reuteri DSM 17938 reduced gastric distension and accelerate gastric emptying. In addition, this probiotic strain seemed to diminish the frequency of regurgitation [96]. In conclusion, certain probiotics may be used to alleviate symptoms of GP such as bloating and delayed gastric emptying and may open new horizons for improving other symptoms of GP. As there is a lack of causal treatment in all pain-related FGIDs, the role of probiotics is increasingly recognized (Table 1), not only for treatment, but also for prevention [93], although evidence for the latter function remains very limited. These data should be interpreted cautiously since they are represented by small-scale low quality, studies that were not controlled or randomized, as well as small studies with pre- and post-measurements. Furthermore, for other indications of probiotic use, recommendations should be limited only to strains with proven efficacy.

Usually, in terms of prebiotics, fiber is generally avoided by individuals with gastrointestinal motility disorders, such as gastroparesis, due to an increased likelihood of exacerbated symptoms. Therefore, fiber needs to be carefully used among those with motility issues for a wide variety of reasons [97]. In this setting, however, prebiotic gum fibers are the best candidates for people with slow motility issues [98]. Finally, particular attention should be paid to antibiotics. Indeed, the pro-motilin macrolide antibiotic erythromycin induces the acceleration of gastric emptying by determining antral contractions and ameliorates gastroduodenal coordination [99,100]. For this reason, this antibiotic is popular for the treatment of gastroparesis in the USA. However, like cisapride, it harbors cardiac side-effects such as QT prolongation, thus causing potentially fatal cardiac arrhythmia [101]. Moreover, further gastrointestinal side effects such as nausea and diarrhea are common [102]. Additionally, erythromycin has possible interactions with drugs that are metabolized by cytochrome P450, such as warfarin, carbamazepine, and theophylline [102]. These factors make use of this antibiotic less convenient. Finally, concern about the development of microbial resistance to this antibiotic following its use as a pro-motility agent is also a growing issue. For this reason, azythromicine is used as a safer and more effective alternative to erythromycin in the treatment of gastroparesis; however, long-term data about its safety and the induction of antibiotic resistance or tachyphylaxis are needed [103].

## 3. Exogenous Infections as Trigger of Gastroparesis

As mentioned above, viral, bacterial, and protozoal infections are among the many possible causes of GP. These infections can damage the ENS and muscles in the stomach, leading to delayed gastric emptying. The clinical characteristics of post-infectious gastroparesis are detailed in Table 2. However, this topic needs further research data.

### 3.1. Viral Infections

The association between viral infections and GP has been reported in the literature. However, data are limited and mainly consist of case reports or case series.

The link between viral infections and gastroparesis was first postulated in the 1990s when Oh and Kim described seven young patients (mean age 27 years), without severe comorbidities, who developed nausea, vomiting, and epigastric pain. These patients underwent GES, which showed delayed gastric emptying after a suspected viral illness. The authors reported that during a mean follow-up of 32.3 months, five out of seven patients experienced a complete resolution of gastroparetic symptoms, and the other two presented considerable improvements in their condition. Remarkably, autonomic neuropathy was found in all three patients who underwent the autonomic function test [104].

Research suggests that a significant proportion, approximately 20%, of idiopathic GP cases can be attributed to a preceding viral infection [105]. Bityutski et al. reviewed the medical records of 143 patients diagnosed with GP, finding that 12 patients (21%) had a prior history of viral syndromes. Interestingly, the authors discovered that all patients with post-viral GP, unlike those with idiopathic disease, reported a gradual improvement of symptoms if they had no hospitalization during the previous 6 months, stable weight, no disability, and remained professionally active [105].

Parkman et al. reported that post-viral etiology can be discovered in up to 23% of cases of idiopathic GP. Epstein–Barr virus (EBV), varicella-zoster virus (VZ), and Cytomegalovirus (CMV) represented the most common triggers of this condition. In this study, post-viral GP was defined as the persistence of dyspeptic symptoms for at least three months following such gastro-intestinal infection [106].

Post-viral GP is classified within the group of post-viral dysfunctions that affect the autonomic nervous system [107]. However, when they occur in the context of dysautonomia, such dysfunctions can be associated with a poor prognosis [108].

Coronavirus disease 2019 (COVID-19) is an infectious respiratory illness caused by the highly contagious severe acute respiratory syndrome coronavirus 2 (SARS-CoV-2), which was first reported in Wuhan, China, in December 2019 [109]. Over the past four years, the COVID-19 pandemic has rapidly spread across the globe, resulting in devastating effects on society. Individuals diagnosed with COVID-19 typically exhibit respiratory symptoms, as the virus primarily attacks the respiratory system [110]. However, gastrointestinal symptoms have also been reported [111]. A meta-analysis of 60 studies that included 4243 COVID-19 patients revealed a pooled prevalence of 18.6% for all gastrointestinal symptoms. These symptoms included anorexia (26.1%), diarrhea (13.5%), nausea/vomiting (9.4%), and abdominal pain (5.7%) [111].

Few case reports in the literature have found an association between COVID-19 and GP. However, the condition is likely to be underestimated, as patients who have been infected or are currently infected with COVID-19 and develop gastroparetic symptoms are not usually tested for GES, given that such symptoms are often self-limiting and resolve on their own.

It was reported that COVID-19 can lead to the development of GP in healthy patients or exacerbate a severe GP flare in pre-existing GP.

Rusch et al. described the case of a healthy 16-year-old female subject who presented with abdominal pain, early satiety, and vomiting. This patient likely had asymptomatic COVID-19 2 months prior to presentation. After investigation of the epidemiologic links, antibody testing, and a clinical course, the patient was diagnosed with post-viral GP due to COVID-19. She was treated with supportive care and prokinetic agents and demonstrated symptom resolution and the near normalization of gastric emptying by the time of her 1-month follow-up [112].

On the other hand, Song et al. reported the case of a 37-year-old female patient with a history of diabetic GP, who developed nausea and vomiting similar to her GP flares. Initially, a clinical response was not observed after treatment with conventional prokinetics. After developing a fever, she was tested and found to be positive for COVID-19. Antibiotic, steroid, and antiviral medications were then administered, resulting in a significant improvement of her symptoms. By day 4 of hospitalization, her fever had subsided, and she was discharged on day 5. The patient reported improvements in her symptoms at a follow-up outpatient gastroenterology visit 2 months later [113].

Kundu et al. reported a case of likely post-viral GP (unknown pathogen) that was effectively treated with mirtazapine. The authors described the case of a 34-year-old woman who developed nausea, vomiting, and weight loss following a viral infection contracted by her children. GES confirmed the diagnosis of GP. Despite treatment using various medications with conventional pro-kinetic agents and numerous anti-emetic drugs, her symptoms persisted and resulted in multiple hospitalizations. The patient experienced a significant reduction in nausea, cessation of vomiting, and improved tolerance to oral intake after taking mirtazapine. The authors concluded that mirtazapine may be effective in treating symptoms associated with non-diabetic and post-viral GP, which are refractory to conventional therapies [114].

Acquired Immunodeficiency Syndrome (AIDS) is a disease caused by the human immunodeficiency virus (HIV), which attacks and weakens the immune system, leaving the body vulnerable to infections and other diseases. CMV is one of the most feared opportunistic infections in HIV-infected individuals, which can cause a range of symptoms including fever, fatigue, and organ damage [115]. Thongpooswan et al. reported the unique case of a 46-year-old female patient with AIDS who developed lumbosacral polyradiculopathy and delayed gastric emptying resulting during CMV infection. The authors point out that the patient was not diabetic, had not undergone surgery, and had no other risk factor for gastroparesis. Therefore, they attributed the development of delayed gastric emptying to the CMV infection. This hypothesis was supported by the gradual improvement of gastric symptoms after the patient started treatment with gancyclovir. Nausea and vomiting resolved after treatment over 8 weeks. CMV infection can cause neuropathy, which may involve both direct cellular infections and/or molecular mimicry and can manifest as diffuse encephalitis (the most common type), ventriculo-encephalitis, myelitis, polyradiculopathy, or mononeuritis multiplex. The development of gastroparesis could have been the result of damage to the enteric nervous system caused by the virus [115].

Enterovirus infection represents another etiology of post-viral GP. Barkin et al. conducted a study to document cases of Enterovirus infection as a possible cause of idiopathic GP. They assessed 11 patients; 9 of these had a history of flu-like symptoms or gastroenteritis and underwent gastric biopsy, which revealed active Enterovirus infection. Eight out of nine patients received treatment with antivirals and/or immune therapies. Follow-up was available only for five patients; four patients showed fast symptomatic improvements [116].

### 3.2. Bacterial Infections

When a bacterial infection affects the digestive system, it can cause symptoms such as diarrhea, abdominal cramps, and vomiting. Bacterial infection can also cause GP. However, data on the topic are very limited. The relationship between bacterial infection and GP is bilateral. Indeed, while bacterial infection can lead to GP, GP can also provoke bacterial overgrowth in the stomach and small intestine (See the Section 2.1).

The main pathogenic bacteria associated with the development of GP are *Salmonella* and *Helicobacter Pylori* (HP), although little evidence exists.

*Salmonella* gastroenteritis is a significant risk factor for the development of dyspepsia, as shown by Mearin et al. In this study, the authors enrolled 1878 adult patients affected by *Salmonella* and demonstrated that at 1-year follow-up, one in seven subjects developed dyspepsia. However, the GET of patients was not evaluated by scintigraphy; the authors merely speculated that dyspeptic symptoms could be associated with delayed gastric emptying caused by the bacterial infection [117].

HP infection is common in humans, affecting around 50% of the world’s population [118,119]. HP is linked to the development of chronic gastritis, peptic ulcers, and gastric cancer, as well GBA disorders [120] such as FD and GP [121,122].

Salicru et al. conducted a multicenter study with 3040 patients with GP and 575,895 controls and revealed that while reactive gastropathy was marginally more prevalent in patients with GP (18.9%) than in the controls (17.0%), the HP gastritis rate was lower in patients with GP compared to that in healthy subjects (5.9% versus 10.8%, respectively). The authors speculated that the low prevalence of HP infection in gastroparetic patients could be due to higher rates of previous eradication or the protective effect of mucosal inflammation against the development of motility disorders. However, the primary limitation of this study was that GP was not defined based on scintigraphic examination but only based on clinical symptoms. This factor represents a significant bias and makes the results unreliable [123].

Since then, the latest evidence has shown that HP infection is associated with GP.

Notably, Huang investigated the correlation between HP infection and diabetic GP in a retrospective setting. In this study, GP was evaluated using a barium X-ray exam. The authors included 163 patients with type 2 diabetes mellitus and 175 non-diabetic controls who were divided into diabetic GP, simple diabetes, non-diabetic GP, and normal groups based on their conditions. The authors found that the HP infection rate was significantly higher in the diabetic GP group (74.6%) compared to the other groups (simple diabetes 51.1%, p < 0.01; non-diabetic GP 57.7%, p < 0.06; normal group 48.0%, p < 0.01) and, interestingly, revealed that patients with diabetic GP showed a significant reduction of gastric emptying in a barium X-ray taken after successful HP eradication [124].

Recently, Liu et al. found, in a mouse model, the pathogenetic mechanism of HP associated with GP; the bacterial infection led to a reduction in the number of Interstitial Cajal Cells (ICCs) and alteration of the ICC network due to a decrease in the level of Stem Cell Factor (SCF), a protein known to activate the proliferation and function of ICCs through the activation of the c-kit receptors [125].

### 3.3. Protozoal Infections

The relationship between human protozoal diseases and gastric emptying has been poorly investigated, with limited data available in the literature on the subject. Malaria and Chagas disease are the only infections that have been assessed in this regard. Existing studies have revealed that patients affected by these infections may present altered gastric emptying, including delayed emptying.

Despite recent efforts and successes in reducing the global burden of malaria, severe malaria still accounted for the majority of the 619,000 reported malaria deaths in 2021 [126]. This disease can manifest as a range of clinical conditions due to the involvement of multiple organs, including cerebral malaria, renal failure, jaundice, and anemia [127,128]. Patients with malaria often experience vomiting, nausea, regurgitation, and epigastric pain [129]. Mohapatra et al. hypothesized that in patients with cerebral malaria (including those in unarousable comas), such gastrointestinal symptoms may be caused by abnormal gastric motility. To test this hypothesis, the authors performed GES with liquid meals in 25 patients with cerebral malaria and in 10 healthy controls and found that the GEt ½ of patients affected by malaria was significantly prolonged compared to the control group (46.5 ± 4.8 and 27.6 ± 5.3 min, respectively, p < 0.001) [130]. It is known that abnormalities in the central nervous system (CNS) can affect the motor functions of the stomach, directly or through the autonomic nervous system. Cerebral malaria is an acute encephalopathy diffusely affecting the CNS. Thus, delayed gastric emptying could be explained by the hypothesis that brain involvement can evoke gastric dysmotility through the CNS and autonomic supply. Additionally, the authors observed an inverse relationship between the Glasgow Coma Scale (GCS) and GEt ½ (patients with lower GCS scores had increased GEt ½). These data strongly support the role of CNS in controlling gut function.

Wilairatana et al. also evaluated gastric emptying in patients with acute uncomplicated malaria using the paracetamol absorption method. The authors found no differences between acute illness and convalescence among the patients and concluded that gastric emptying was not altered in acute uncomplicated falciparum malaria [131]. However, the reliability and usefulness of the paracetamol absorption method for studying gastric motility remains a topic of debate [132].

Chagas disease is a protozoal infection caused by *Trypanosoma cruzi*. This disease is endemic in Latin American countries and has been disseminated to non-endemic regions on five continents through human migrations, thus representing a significant global health challenge [133].

Chagas infection can progress to a chronic disease that affects the nervous, heart, and digestive systems [134]. The manifestation of the gastrointestinal tract involves the dilation of the viscera, including the megacolon and megaesophagus [134], as well as motor disorders.

It was documented that patients affected by Chagas disease may have altered gastric emptying, which can be delayed or accelerated.

Chinzon et al. assessed the GET of a semi-solid diet via real-time ultrasonography in patients with Chagas megaesophagus compared to non-Chagas controls and determined that GET was significantly increased in the megaesophagus group compared to the control group (192.9 ± 32.7 versus 129.0 ± 29.6 min, respectively, p < 0.001) [135]. The authors stratified patients with megaesophagus in three grades according to the classification system by Rezende et al. [136]. Interestingly, the authors found that the gastric emptying delay was not affected by the entity of megaesophagus dilatation nor by the duration of dysphagia. Delayed gastric emptying in Chagas disease can be explained by damage to the excitatory motor neurons in the ENS caused by the protozoa.

Troncon et al. compared the GES T ½ of 16 patients with Chagas disease including radiographic evidence of esophageal and/or colonic involvement in 18 healthy volunteers and revealed that the GES T ½ of liquid meal in chagasic patients was significantly lower than that in the controls (5.6 ± 3. 7 vs. 11.4 ± 5.5 min, respectively, p < 0.01). Interestingly, the authors found that the time of arrival of the meal to the proximal small bowel was also significantly shorter in patients than in the controls, while observing no differences in the time of the meal’s arrival to the distal small intestine. The authors hypothesized that accelerated gastric emptying was likely caused by the destruction of the inhibitory neural pathways acting on the proximal stomach due to the parasite, resulting in defective gastric accommodation [137].

## 4. Conclusions and Future Perspectives

GP is a disorder that severely impacts the normal functioning of the stomach, leading to delayed gastric emptying and a range of unpleasant symptoms, such as nausea, vomiting, and abdominal pain. Furthermore, the relationship between symptoms and the severity of gastric emptying tests remains poorly understood [29].

Despite its significant impact on patients’ quality of life, the underlying causes of GP remain unclear. However, emerging evidence suggests that microbiota dysbiosis may play a significant role in the development and progression of this condition.

Future research is needed to better understand the correlation between GP and microbiota dysbiosis, including investigating the relevant causal relationships and developing interventions to restore a healthy microbiota in individuals with GP. The development of new diagnostic tools to detect microbiota dysbiosis in individuals with GP may also improve diagnosis and treatment outcomes. Several lines of evidence have demonstrated the pivotal role of the gut microbiome in GI dysmotility [138]. A major challenge in studying the gut microbiota is translating and applying data to physiologically relevant mechanisms. To overcome this challenge, we could isolate specific bacterial strains or analyze how they are conditioned by specific macronutrients commonly found in humans and use the information obtained to clarify relevant biomarkers. These biomarkers could then be used to find the ideal treatment for GI dysmotility disorder. Additionally, due to the obscurity of small intestinal microbiome research, more attention should be paid to the pathogenesis of SIBO in GI dysmotility disorders.

The association between SIBO and GP/FD remains unclear, as does the gastrointestinal segment most relevant in this disease’s pathogenesis. Gastric bacterial overgrowth remains, to date, a rare condition [139] that needs to be further elucidated. According to data in the scientific literature, the most studied segment in this setting is currently the small intestine, particularly the duodenum.

Currently, the diagnosis of GP is based on a combination of symptoms, physical examinations, and gastric emptying studies. However, these tests do not always provide a definitive diagnosis, and it can be difficult to differentiate GP from other gastrointestinal disorders with similar symptoms such as FD. The use of new diagnostic tools that can detect microbiota dysbiosis in individuals with GP may provide a more accurate and reliable diagnosis, allowing for more targeted treatment approaches. Additionally, identifying specific microbial imbalances associated with GP may enable the development of personalized treatment plans that target underlying dysbiosis, such as probiotics, antibiotics, or fecal microbiota transplantation.

Infections, including viral, bacterial, and protozoal inflections, can also play a significant role in the development of GP. The effective treatment of infections is crucial to improving the symptoms and quality of life in individuals with GP caused by any of these factors. In most cases, post-viral delayed gastric emptying is a self-limiting condition that resolves on its own. However, despite being a widespread clinical factor potentially linked to the dyspeptic symptoms of many viral infections, post-viral GP is very likely to be vastly underestimated and requires further evaluation.

Further studies are also needed to confirm HP’s role as a triggering pathogen for GP in clinical practice when considering recent experimental evidence, mainly in gastric emptying studies. GP associated with protozoal infections affects vulnerable individuals in developing countries who are often in a critical condition. Additional studies are needed to better define the clinical role of gastric motility disorders in the context of such systemic infections.

In conclusion, the relationship between exogenous microorganisms, microbiota, and GP is an iceberg that still hides most of its secrets. We await new evidence to update our notions of the basis of this complex interplay.

## Figures and Tables

**Table 1:** Evidence regarding the use of probiotics in gastroparesis and functional gastrointestinal disorders.

Author, Year	Study Design	Probiotic Studied	Number of Patients	Remarks
Wang, 2012 [94]	Crossover placebo-controlled clinical trial	*L. paracasei* 33, *L. fermentum* and *L. acidophilius*	15	The multi-strain Lactobacillus capsule is safe and accelerates gastric emptying in healthy adults older than 40 years of age. It may become a therapeutic approach in future trials for pathological gastric emptying delay, especially in diabetic gastroparesis
Ringel-Kulka, 2011 [95]	Double-blind, placebo-control clinical trial	*Lactobacillus acidophilus* and *Bifidobacterium lactis*	60	Administration of these probiotics twice a day improved symptoms of bloating in patients with FGIDs
Indrio, 2011 [96]	Randomized controlled trial	*L. reuteri* DSM 17938	42	In infants with gastroesophageal reflux disease, *L. reuteri* DSM 17938 reduced gastric distension and accelerated gastric emptying

**Table 2:** Data highlighting the typologies of post-infective gastroparesis.

	Virus 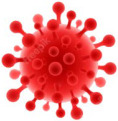	Bacteria 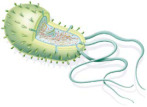	Protozoa 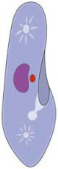
Pathogens	Epstein–Barr virus (EBV), Varicella–zoster virus (VZ) Cytomegalovirus (CMV), Coronavirus disease 2019 (COVID-19), Enterovirus	*Salmonella* gastroenteritis, *Helicobacter pylori* (HP)	*Trypanosoma cruzi*, *Plasmodium falciparum*
GP pathogenesis	Damage to the autonomic nervous system	In the case of HP infection, there is a reduction in Interstitial Cajal Cells (ICCs) (mouse model)	Damage to the central nervous system and autonomic system (excitatory and/or inhibitory neurons)
GP clinical aspects	Mild symptoms, does not cause serious disabilities. In the case of COVID-19 infection, reported flare with underlying known GP	Mild symptoms	Delayed and accelerated gastric emptying. Severe symptoms in frail patients
Clinical course	Data lacking, self-limiting, good prognosis	Data lacking. Excellent prognosis	Data lacking. Severe prognosis, frequent association with megaesophagus in Chagas disease, and coma in cerebral malaria
Treatment	Supportive care, prokinetics, antiviral. In cases refractory to conventional drugs, reported efficacy of mirtazapine	Supportive care, prokinetics. Reduction of gastric emptying time after successful HP eradication	Supportive care, prokinetics, antiprotozoal drugs

## Data Availability

Not applicable.
